# Using a decision tree algorithm to distinguish between repeated supra-therapeutic and acute acetaminophen exposures

**DOI:** 10.1186/s12911-023-02188-2

**Published:** 2023-06-01

**Authors:** Omid Mehrpour, Christopher Hoyte, Samaneh Nakhaee, Bruno Megarbane, Foster Goss

**Affiliations:** 1 Michigan Poison & Drug Information Center, Wayne State University School of Medicine, Detroit, MI USA; 2 School of Medicine, University of Colorado, Aurora, CO USA; 3 Medical Toxicology and Drug Abuse Research Center (MTDRC), Birjand University of Medical Sciences (BUMS), Birjand, Iran; 4 Department of Medical and Toxicological Critical Care, Lariboisière Hospital, INSERM UMRS, University of Paris, Paris, 1144 France; 5 Department of Emergency Medicine, University of Colorado School of Medicine, Aurora, CO USA

**Keywords:** Decision Tree, APAP, Repeated supra-therapeutic ingestion, Acute acetaminophen poisoning, NPDS

## Abstract

**Background:**

This study aimed to compare clinical and laboratory characteristics of supra-therapeutic (RSTI) and acute acetaminophen exposures using a predictive decision tree (DT) algorithm.

**Methods:**

We conducted a retrospective cohort study using the National Poison Data System (NPDS). All patients with RSTI acetaminophen exposure (n = 4,522) between January 2012 and December 2017 were included. Additionally, 4,522 randomly selected acute acetaminophen ingestion cases were included. After that, the DT machine learning algorithm was applied to differentiate acute acetaminophen exposure from supratherapeutic exposures.

**Results:**

The DT model had accuracy, precision, recall, and F1-scores of 0.75, respectively. Age was the most relevant variable in predicting the type of acetaminophen exposure, whether RSTI or acute. Serum aminotransferase concentrations, abdominal pain, drowsiness/lethargy, and nausea/vomiting were the other most important factors distinguishing between RST and acute acetaminophen exposure.

**Conclusion:**

DT models can potentially aid in distinguishing between acute and RSTI of acetaminophen. Further validation is needed to assess the clinical utility of this model.

## Introduction

Acetaminophen is a commonly used antipyretic and analgesic drug available over the counter [1]. In adults, the safe dose of acetaminophen is between 325 and 650 mg every 4–6 h, with a maximum of 4 g per day [2]. Overdose of acetaminophen can be severe, leading to liver failure or even death [3]. Acetaminophen poisoning is one of the most common causes of acute liver failure in the US and worldwide [4]. Although most patients with acetaminophen poisoning have mild-to-moderate consequences, 29% of patients with acetaminophen-induced liver failure require a liver transplant and have a mortality rate of 28% [5].

Most commonly, acetaminophen overdose occurs after ingesting acetaminophen, within 8 h or less, in amounts that can cause toxicity (e.g., > 4 g/24 hours). Although hepatotoxicity may occur from acute or repeated acetaminophen ingestion, mortality from acetaminophen poisoning is uncommon​​ (death occurred in 0.3% of cases treated with N-acetylcysteine). Chronic exposure is termed repeated supra-therapeutic ingestion (RSTI) to separate it from regular therapeutic use. In adults, RSTI of acetaminophen occurs when the intake of acetaminophen exceeds 8 h, resulting in a cumulative dose of more than 200 mg/kg/day (or 10 g/day, whichever is less) within 24 h or more than 150 mg/kg/day (or 6 g/day, whichever is less) within 48 h [6, 7]. In addition, for children under six years of age, RSTI is the repeated consumption of acetaminophen of more than 100 mg/kg/day for 72 h [7].

Contrasting with acute exposure cases with known ingestion time, in RSTI, the acetaminophen nomogram-based approach is not applicable. There are few studies and guidelines vary in what constitutes RSTI and when to start N-acetylcysteine. Typically, N-acetylcysteine is recommended in patients with alanine aminotransferase (ALT) greater than 50 IU/L or serum acetaminophen concentration greater than 20 mg/L (132 µmol /L) [8, 9]. Daly et al. studied 249 RSTI of acetaminophen and found that patients with AST < 50 U/L and acetaminophen concentrations less than 10 mg/L had a lower risk of hepatotoxicity [10, 11]. As management of RSTI and acute acetaminophen ingestion may differ, it is crucial to differentiate between these two overdose presentations.

Machine learning (ML) is a new approach that has recently been used in medicine for disease diagnosis, treatment decisions and prognosis [12]. ML uses algorithms and mathematical methods to identify patterns and relationships between variables and to contribute to the predictability of the target variable [13]. Supervised and unsupervised learning are two main ML approaches to classifying patients and creating risk detection models [14]. DT models are one of the most commonly used ML algorithms to classify medical data to inform appropriate medical decision making [15–18]. This study uses a DT model to help distinguish between RSTI and acute acetaminophen ingestion using clinical and laboratory characteristics from the National Poison Data System (NPDS).

## Methods

### Study design

This was a retrospective cohort study using NPDS, a de-identified national repository for poison control center data used by the American Association of Toxic Control Centers (AAPCC). The AAPCC maintains case records of self-reported information collected by callers during exposure management and poison information calls managed by the 55 poison control centers across the United States. Poison control centers submit de-identified case data to the NPDS after providing poison exposure management. As soon as information regarding this file is available, it is uploaded to the NDPS and recorded by poison center staff.

The NPDS includes clinical effects and interactions with the agent, duration of effect, end-organ effects, chronicity, demographic data (age, sex, and weight), administration sites, clinical findings, exposure information, and categorical laboratory findings. Each of these was included in the model. In addition, we defined “related to the exposure” to be appropriate when: (1) the timing of clinical effect is consistent with the exposure recorded (clinical effects recorded matches the half-life of toxicant in the body); (2) the severity of the clinical effect is consistent with the reported exposure; (3) the clinical effect is consistent with the expected toxicity of the substance; and (4) a physician assessed the relationship’s clinical significance.

Last, correlational analyses do not necessarily indicate causality. Clinical effects are not related to exposure if they existed before the exposure and did not increase or worsen as a result of the exposure or if the effect can be attributed to an alternative cause. All methods were carried out in accordance with relevant guidelines and regulations.

While most cases are “closed” within a few hours of the initial contact, some exposures are followed to obtain the patient’s medical outcome and may remain open for months. Follow-up calls provide a proven mechanism for monitoring the appropriateness of management recommendations, enabling continual updates of case information, augmenting patient guidelines, providing poison prevention education, and obtaining final medical outcomes to make the data collected as accurate and complete as possible.

### Study population

All patients with RSTI of acetaminophen (n = 4,522) were included between January 2012 and December 2017. In addition, 4,522 randomly selected acute acetaminophen ingestion cases were included. Criteria for exclusion were missing demographic data and clinical findings. We assumed that duplicated data was not included.

We obtained de-identified data from NPDS. Based on the Colorado Multiple Institutional Review Board (COMIRB) on Human Subjects Protection guidelines and procedures, the analysis of NPDS data for this research did not meet the criteria for human subjects research according to the 45 Code of Federal Regulations (CFR) 46.101(b). Therefore, this study was determined to be exempt (COMIRB#: 22-1088).

### ML-based approach

We used a DT model using Statistical Package for the Social Sciences (SPSS) 26 and Python (version 3.9) for distinguishing between RSTI and acute acetaminophen ingestion using clinical and laboratory characteristics extracted from the NPDS. The DT model uses split criteria to divide an end node and set a tree with a rate for each predictor variable [19]. It divides the data into binary parts and builds a binary tree based on them so that two edges come out of each inner node, and the resulting trees are pruned. This algorithm is used to create regression and classification trees. A DT model evaluates the variable that best divides the data [20] using the Gini index criterion to decide how to select tree nodes. The Gini Cost Function shows how pure the nodes are, wherein the purity of the node refers to the degree to which the training data assigned to each node is combined. The division continues until the node has the minimum number of training samples or exceeds the maximum depth of the tree. The root node is the most critical variable that starts the decision tree graph. It is the variable that best divides the data in a DT approach. Intermediate nodes are those in which variables are estimated but not the ultimate nodes in which predictions are formed.

Similar to other ML methods, standard performance measures were used to measure the performance of the DT model, including precision, recall, accuracy, F1 score, and confusion matrices. A confusion matrix was generated with all the required information to measure specificity, accuracy, and sensitivity [13].

### Statistical analysis

Analysis was performed using Python 3.9 and SPSS 26. Chi-square and student t-tests were used to compare data between groups. A P-value of < 0.05 was determined to be statistically significant.

## Results

**Fig. 1:**
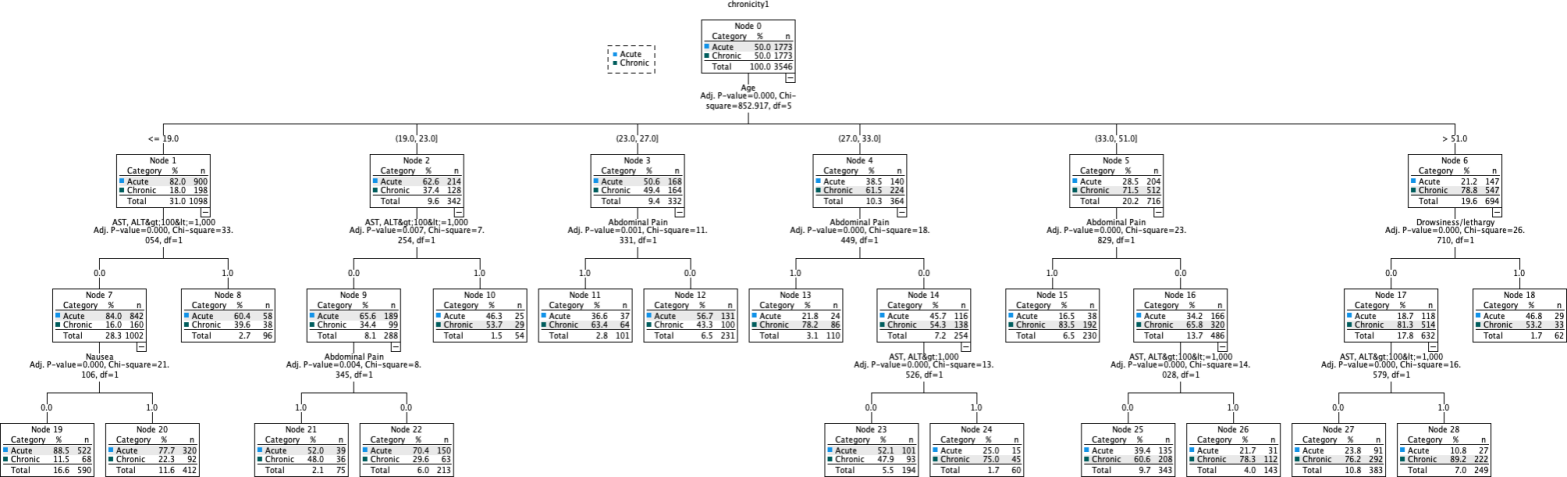
DT diagram for predicting repeated supra-therapeutic ingestion (RSTI) versus acute acetaminophen exposure. Values shown are in percentages

In total, 4,522 patients with RSTI of acetaminophen were included. The baseline and clinical characteristics of patients were compared between two groups, and their results are presented in Table 1. The DT model produced in this study was 29 nodes in size, with 17 leaves and four levels (Fig. 1).

**Table 1:** Characteristics of patients with acute exposure vs. supra-therapeutic ingestions (RSTI)

Variable		Acute exposure (n = 4,522)	RSTI (n = 4,522)	p-value
Sociodemographic	Age (mean ± SD)	23.7 ± 14.6	40.3 ± 18.4	< 0.001
	Gender (male)	3,276	2,950	< 0.001
Medical outcome	Major effect	320	813	< 0.001
	Moderate effect	1,219	1,987	
	Minor effect	2,983	1,722	
Gastrointestinal findings	AST, ALT levels >1000	401	1288	< 0.001
	1000 > AST, ALT > 100	466	1,415	< 0.001
	Increased bilirubin	114	422	< 0.001
	Abdominal Pain	1,076	1,522	< 0.001
	Nausea	2,048	1,825	< 0.001
	Vomiting	2,349	1,577	< 0.001
	LFT abnormality - other	108	275	< 0.001
	Anorexia	6	34	< 0.001
Neurological findings	Confusion	64	135	< 0.001
	Drowsiness/lethargy	334	182	< 0.001
	Coma	45	37	0.22
Cardiovascular and respiratory findings	Hypotension	49	102	< 0.001
	Conductance disturbance	22	12	0.08
	Pulmonary edema	0	3	0.12
Coagulation findings	Prolonged PT/INR	282	748	< 0.001
	Coagulopathy (other)	70	257	< 0.001
	Cytopenia	4	32	< 0.001
	Other bleeding	7	16	0.046
Renal findings	Oliguria/anuria	13	43	< 0.001
	Renal failure	15	81	< 0.001
	Creatinine increased	47	231	< 0.001
Dermatology findings	Erythema/flushing	15	45	< 0.001
Endocrinology findings	Hypoglycemia	10	37	< 0.001
Laboratory findings	Acidosis	97	238	< 0.001
	Increased anion gap	69	97	0.017
	Electrolyte abnormality	133	201	< 0.001
Miscellaneous findings	Diaphoresis	14	29	0.016
	Fever/hyperthermia	8	19	0.026
NAC-IV		2,679	3,090	< 0.001
NAC-PO		427	522	0.003
Phytonadione		48	128	< 0.001

AST: Aspartate transaminase, ALT: Alanine transaminase, LFT: Liver function test, PT: prothrombin time, INR: international normalized ratio, NAC: N-acetylcysteine.

**Fig. 2:**
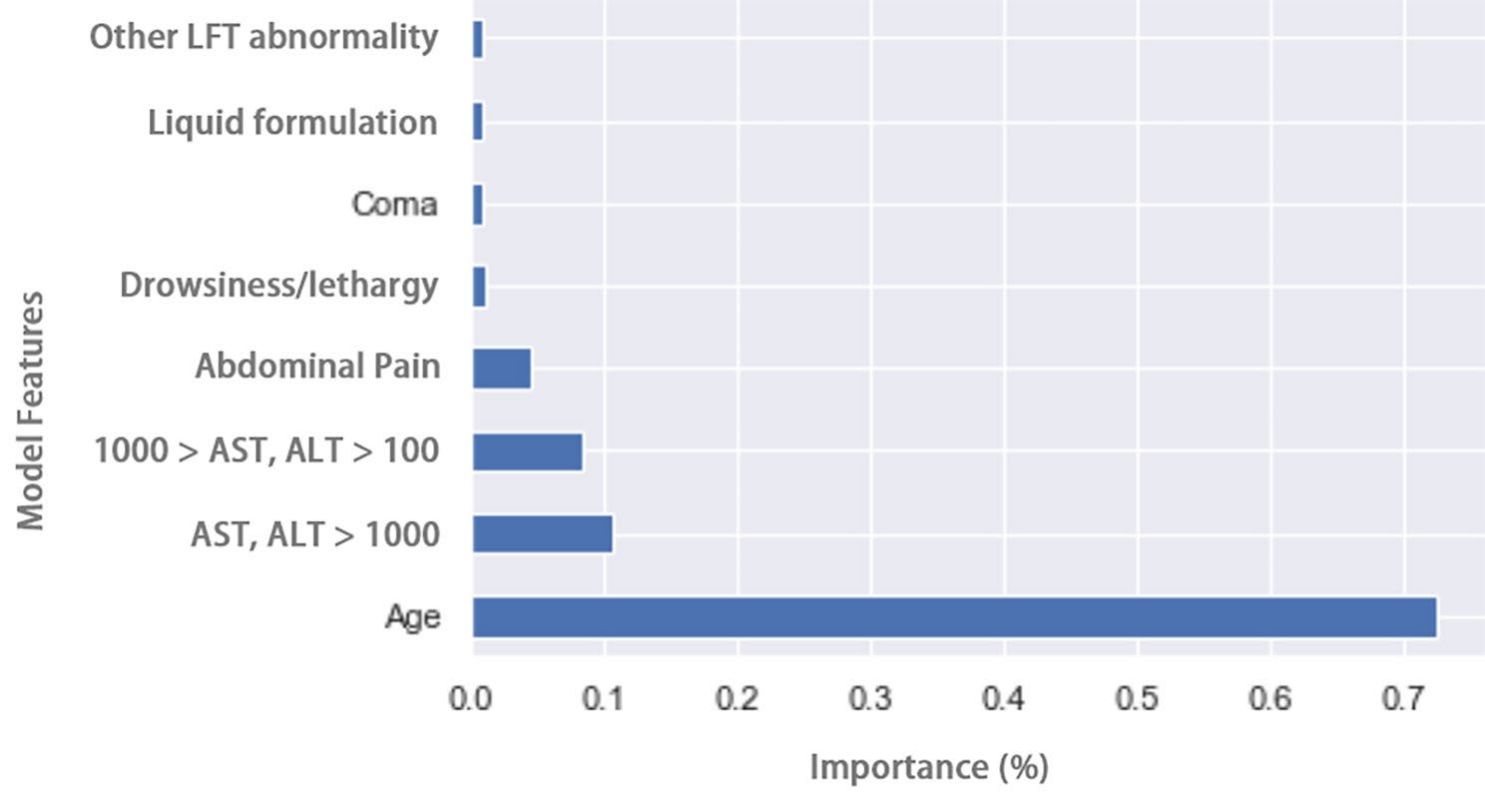
Feature importance based on the DT model

Age, followed by serum aminotransferase concentrations, abdominal pain, and drowsiness/lethargy, were the most important factors in distinguishing between RSTI and acute acetaminophen exposures (Fig. 1). The rules derived from the DT are shown in Table 2. In Fig. 2, we evaluated important signs and symptoms that affect the DT model performance (i.e., feature importance) in the classification task. Serum aminotransferase concentrations, abdominal pain, nausea/vomiting, and drowsiness/lethargy contributed most to classifying acute and RSTI, respectively.

**Table 2:** The 17 rules extracted from the DT model to distinguish between acute and repeated supra-therapeutic (RST) acetaminophen exposures

**The patient is more likely to have acute acetaminophen exposure IF:**			
33 > age > 27 years	Abdominal pain is absent	AST, ALT < 1000	52.1% likelihood
27 > age > 23 years	Abdominal pain is absent		56.7% likelihood
23 > age > 19 years	AST, ALT < 100 or >1000	Abdominal pain is absent	70.4% likelihood
23 > age > 19 years	AST, ALT < 100 or >1000	Abdominal pain is present	52.0% likelihood
age < 19 years	1000 > AST, ALT > 100		60.4% likelihood
age < 19 years	AST, ALT < 100 or >1000	Nausea is present	77.7% likelihood
age < 19 years	AST, ALT < 100 or >1000	Nausea is absent	88.5% likelihood
**The patient is more likely to have RSTI of acetaminophen IF**:			
age > 51 years	Drowsiness/lethargy is present		53.2% likelihood
age > 51 years	Drowsiness/lethargy is absent	1000 > AST, ALT > 100	89.2% likelihood
age > 51 years	Drowsiness/lethargy is absent	AST, ALT < 100 or >1000	76.2% likelihood
51 > age > 33 years	Abdominal pain is absent	1000 > AST, ALT > 100	78.3% likelihood
51 > age > 33 years	Abdominal pain is present		83.1% likelihood
51 > age > 33 years	Abdominal pain is absent	AST, ALT < 100 or >1000	60.6% likelihood
33 > age > 27 years	Abdominal pain is absent	AST, ALT > 1000	75.0% likelihood
33 > age > 27 years	Abdominal pain is present		78.2% likelihood
27 > age > 23 years	Abdominal pain is present		63.4% likelihood
23 > age > 19 years	1000 > AST, ALT > 100		53.7% likelihood

ALT, alanine aminotransferase; AST, aspartate aminotransferase; RSTI, repeated supra-therapeutic ingestion

**Fig. 3:**
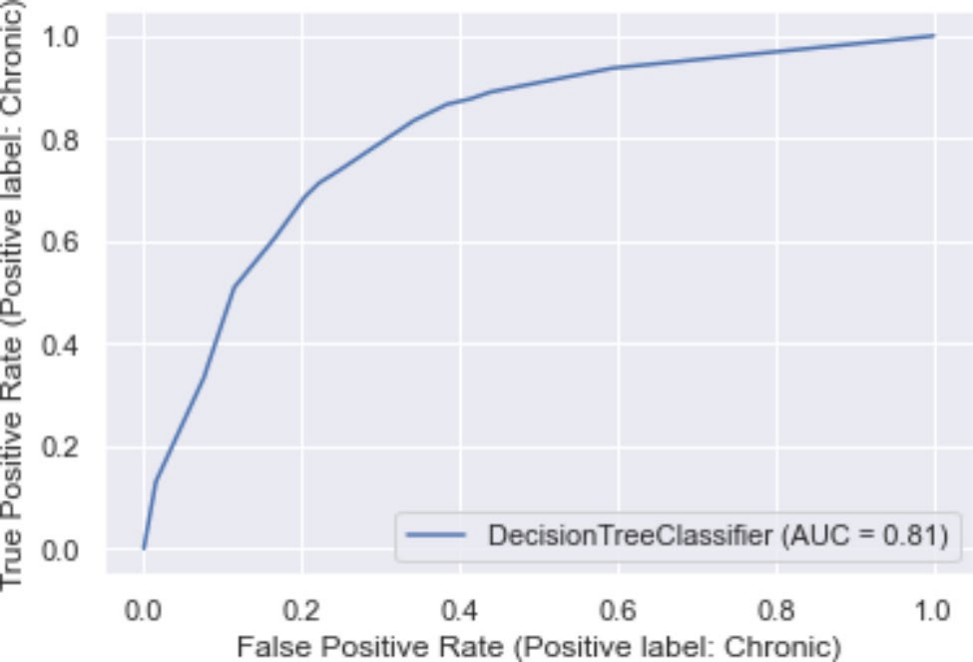
Receiver Operating Curve (ROC) for the DT model

The DT model had an accuracy, precision, recall, and F1-score of 0.75 each in the cross-validation method (Table 3). Confusion matrices are shown in Table 4. The model could correctly predict 3,361 cases of acute acetaminophen poisoning and 3,381 RSTIs. Figure 3 shows the ROC curve for the DT model (AUC = 0.81).

**Table 3:** Characteristics of the DT model in the acute and RSTI acetaminophen poisoning

Labels	Acute exposure	RSTI exposure	Average	Weighted average
**Precision**	0.75	0.74	0.75	0.75
**Recall**	0.74	0.75	0.75	0.75
**F1-score**	0.74	0.75	0.75	0.75
**Accuracy**	-	-	0.75	-

RSTI, repeated supra-therapeutic ingestion

**Table 4:** Confusion matrix of DT in acute and RSTI acetaminophen poisoning

Prediction	Acute exposure	RSTI exposure
True		
**Acute exposure**	3,361	1,161
**RSTI exposure**	1,141	3,381

RSTI, repeated supra-therapeutic ingestion

## Discussion

We assessed the efficacy and accuracy of a DT model to distinguish acute and RSTI of acetaminophen using retrospective large-scale NPDS dataset analysis. Our DT model had acceptable characteristics and could correctly predict 3,361 cases of acute acetaminophen poisoning and 3,381 RST exposures. These findings show promise in using ML to help differentiate acute ingestions from RSTIs.

In our DT model, the most relevant predictive variable was age. This result is unsurprising given that elderly patients are more likely to use acetaminophen as an analgesic due to various ailments, raising the possibility of poisoning due to RSTI. Our study found that most RSTI exposures tend to produce aminotransferase concentrations between 100 and 1,000 IU/L or greater than 1,000 IU/L. Prior studies have found that all cases with RSTI who developed hepatotoxicity were found to have abnormal ALT (more than 50 IU/L) [8] and more likely to have encephalopathy requiring renal replacement therapy, mechanical ventilation, or death [21]. The risk of liver failure has also been seen in patients with RSTI compared to acute overdose cases [22]. This may be partly due to the delayed treatment of RSTI patients as compared to acute acetaminophen exposure cases, which typically undergo treatment within eight hours of exposure [23, 24].

Our data also showed that acidosis is another important variable in RSTI exposures. Anion gap metabolic acidosis can occur in patients with acetaminophen overdose-induced hepatotoxicity and occasionally precedes hepatic injury [25]. In addition, studies showed that electrolyte abnormalities due to an acetaminophen overdose correlate with metabolic acidosis [26, 27].

We also found that patients with RSTI of acetaminophen had increased creatinine concentrations and prolonged PT/INR compared to the acute group. Other studies have shown that patients with RSTI have increased creatinine concentrations, and serum INR had a significant association with hepatotoxicity. All patients who died or had a liver transplant showed increased creatinine concentrations [23]. It is possible that people with RSTI had an acute illness (e.g., hepatitis, dehydration) causing the acute kidney injury and minor elevation in transaminases. The dataset does not include information about the prior or concomitant illness that likely caused the kidney injury.

Our findings also showed that gastrointestinal manifestations, including abdominal pain and nausea/vomiting, might help differentiate acute and RSTI of acetaminophen exposures. Previous literature characterized nausea, vomiting, anorexia, diaphoresis, malaise, pallor, and lethargy during the first 24 h post-acute ingestion [28, 29]. Many authors reported gastrointestinal manifestations in acetaminophen poisoning cases at presentation or during N-acetylcysteine administration [30–34]. The prevalence of gastrointestinal manifestations in hospitalized patients with acute acetaminophen overdose was 76.6% [35]. A latency time of more than 8 h and an ingestion dose of 10 g can predict a higher prevalence of gastrointestinal manifestations. In addition, patients with gastrointestinal manifestations suffered more liver and kidney damage [35].

Interestingly, we found that the patients with RSTI had more diaphoresis and erythema than acute acetaminophen exposure. It is possible that N-acetylcysteine could be contributory, but more investigation is required to understand this finding.

Our study has limitations. The retrospective design of the current study may have resulted in biases due to confounding factors. Future studies will be required to assess and validate our models’ performance. Other limitations include insufficient documentation during poison center calls or possible transcription errors. Since NPDS provides data that has been fully anonymized, we assumed duplicate data was not included but could not determine precisely.

Also, this study included cases of acetaminophen exposure between January 2012 and December 2017. To enhance predictive accuracy, future studies are needed to include data from recent years, larger data sets, and more sophisticated modeling methods considering different variables such as the acetaminophen formulation taken and co-ingested drugs. The doses of acetaminophen and time to N-acetylcysteine administration are the most important considerations that cannot be determined from NPDS data. There is no way to determine an acetaminophen concentration, no determination of time from ingestion to time to N-acetylcysteine, and most importantly, no way to determine the duration of exposure other than the standard definitions. The NPDS provides the coded abnormalities in the course of hospitalization, not at arrival at the hospital. So, this point limits the generalizability of ML applications at the patient’s arrival and specific course of hospitalization. In addition, the NPDS-coded data are limited and do not provide enough information regarding the time course of poisoning. Future studies considering other data sources that include AST/ALT and acetaminophen concentrations at presentation would help to address the question. Additionally, data regarding RSTI management, including antidotes, would be useful to collect to be able to evaluate the optimal treatment to provide.

## Conclusion

A DT model can assist in distinguishing acute and RSTI of acetaminophen exposures. The most important distinguishing factors are age, serum aminotransferase concentrations, abdominal pain, nausea/vomiting, and drowsiness/lethargy. Clinical validations will be necessary before use in clinical settings.

## Data Availability

The datasets used and/or analyzed during the current study are available from the corresponding author upon reasonable request.

## Declarations

### Disclaimers

AAPC supports the NPDS, which includes the data of all self-reporting patients collected by callers during poison information calls and exposure management by the country’s PCCs. However, NPDS data do not reflect the total amount of exposure to a particular substance. Additional exposure to PCC can therefore be under-reported. Accordingly, NPDS data should not confirm poisoning or overdoses, and the AAPCC does not fully verify the accuracy of each report. Therefore, the results based on NPDS data do not necessarily reflect the AAPCC’s opinion.
